# Activation mechanisms of monocytes/macrophages in adult-onset Still disease

**DOI:** 10.3389/fimmu.2022.953730

**Published:** 2022-08-26

**Authors:** Hiroto Tsuboi, Seiji Segawa, Mizuki Yagishita, Hirofumi Toko, Fumika Honda, Ayako Kitada, Haruka Miki, Ayako Ohyama, Shinya Hagiwara, Yuya Kondo, Isao Matsumoto, Takayuki Sumida

**Affiliations:** Department of Rheumatology, Faculty of Medicine, University of Tsukuba, Ibaraki, Japan

**Keywords:** adult-onset still disease, monocytes/macrophages, inflammasome, placenta-specific 8, IL-1β, IL-18

## Abstract

Adult onset Still disease (AOSD) is a systemic inflammatory disorder characterized by skin rash, spiking fever, arthritis, sore throat, lymphadenopathy, and hepatosplenomegaly. Although the etiology of this disease has not been fully clarified, both innate and acquired immune responses could contribute to its pathogenesis. Hyperactivation of macrophages and neutrophils along with low activation of natural killer (NK) cells in innate immunity, as well as hyperactivation of Th1 and Th17 cells, whereas low activation of regulatory T cells (Tregs) in acquired immunity are involved in the pathogenic process of AOSD. In innate immunity, activation of monocytes/macrophages might play central roles in the development of AOSD and macrophage activation syndrome (MAS), a severe life-threating complication of AOSD. Regarding the activation mechanisms of monocytes/macrophages in AOSD, in addition to type II interferon (IFN) stimulation, several pathways have recently been identified, such as the pathogen-associated molecular patterns (PAMPs) and damage-associated molecular patterns (DAMPs)-pattern recognition receptors (PRRs) axis, and neutrophil extracellular traps (NETs)-DNA. These stimulations on monocytes/macrophages cause activation of the nucleotide-binding oligomerization domain, leucine-rich repeat, and pyrin domain (NLRP) 3 inflammasomes, which trigger capase-1 activation, resulting in conversion of pro-IL-1β and pro-IL-18 into mature forms. Thereafter, IL-1β and IL-18 produced by activated monocytes/macrophages contribute to various clinical features in AOSD. We identified placenta-specific 8 (PLAC8) as a specifically increased molecule in monocytes of active AOSD, which correlated with serum levels of CRP, ferritin, IL-1β, and IL-18. Interestingly, PLAC8 could suppress the synthesis of pro-IL-1β and pro-IL-18 *via* enhanced autophagy; thus, PLAC8 seems to be a regulatory molecule in AOSD. These findings for the activation mechanisms of monocytes/macrophages could shed light on the pathogenesis and development of a novel therapeutic strategy for AOSD.

## Introduction

Adult onset Still disease (AOSD) is a rare systemic inflammatory disorder of unknown etiology that occurs at the age of 16 years or older and is characterized by skin rash, spiking fever, arthritis, sore throat, lymphadenopathy, and hepatosplenomegaly (1–3).

A previous nationwide epidemiologic survey of adult Still disease (ASD) in Japan revealed an estimated prevalence of ASD of 3.9 per 100,000 (4). Analysis of 169 ASD patients showed a mean age at onset of 46 years, and only 8 patients (4.8%) developed ASD when they were aged younger than 16 years, whilst the other 158 patients (95.2%) had AOSD. Of the 146 patients with available data on the clinical course, 58 (39.7%) and 50 (34.2%) patients showed monocyclic and polycyclic systemic patterns, whilst 15 (10.3%) and 23 (15.8%) patients showed monocyclic and polycyclic systemic patterns with chronic articular involvement, respectively. Regarding the complications, disseminated intravascular coagulation (DIC) and macrophage activation syndrome (MAS), which are severe life-threating complications of AOSD, were noted in 8 (6.3%) and 19 (15.0%) of 127 patients, respectively. At the last medical examination, 145 of 164 patients (88.4%) had achieved remission, whilst 66 of 169 patients (39.1%) experienced relapse during the observation period. Importantly, lymphadenopathy and MAS were significantly associated with increased risk of relapse. Regarding treatment, oral glucocorticoids were administered to 96% of the patients, whilst methotrexate and biologic agents were administered to 41% and 16% of the patients in that survey, respectively (4). In addition, a recent comprehensive systematic literature review (5) including the nationwide epidemiologic survey in Japan described above (4), showed that the estimated prevalence of AOSD was between 0.73 and 6.77 per 100,000 individuals, and for clinical course, monocyclic systemic/self-limited was 21.1-64.3%, polycyclic systemic/intermittent was 9.3-50.0%, and chronic articular was 11.9-55.6%, respectively. The review also reported that 1.7-23.5% of patients with AOSD developed MAS, and the mortality rate of patients with AOSD was 2.3-16% (5). From these findings, prevention of relapse and MAS seem to be the current unmet medical needs in the management of AOSD.

Intravenous tocilizumab (TCZ), a monoclonal antibody against the IL-6 receptor, has been officially approved for AOSD in Japan on the basis of promising results from a double-blind, randomized, placebo-controlled phase-III trial (6). In that trial, treatment responses such as American College of Rheumatology (ACR) 20 and ACR50 in the TCZ group were double those in the placebo group, and TCZ had a significantly stronger glucocorticoid-sparing effect than that of the placebo; moreover, the TCZ interval could be prolonged after disease control in several patients without flare (6). On the other hand, the possibility that TCZ could trigger the development of MAS in AOSD patients has been proposed (7). Although TCZ could be a hopeful treatment strategy against the prevention of relapse in AOSD, which is one of the unmet medical needs in the management of AOSD, TCZ does not seem to be a complete solution for the inhibition of MAS. For another promising biological agent, anakinra which is a recombinant human IL-1 receptor antagonist also has been examined for treatment of AOSD, while it has not been officially approved for AOSD in Japan. A systematic literature review, in which 15 manuscripts (one open randomized multicenter trial and 14 observational single-arm retrospective studies) were analyzed, showed that the majority of AOSD patients treated with anakinra could achieve a complete remission also in monotherapy, and the treatment with anakinra was associated with an important corticosteroids-sparing effect (8). Interestingly, a single center experience and systematic literature review clarified that the majority but not all of pediatric MAS patients associated with systemic juvenile idiopathic arthritis (sJIA) or autoinflammatory diseases (46 out of 50 reported cases) achieved remission by treatment with anakinra (9). Thus, revealing the accurate and comprehensive mechanisms for activation of monocytes/macrophages is necessary for the development of a new radical therapeutic strategy against AOSD complicated with MAS.

In this review, we survey the pathogenic process of AOSD, including the genetic background, triggers, innate and acquired immune systems, high production of ferritin, and proinflammatory cytokines. In particular, we focus on the activation mechanisms of monocytes/macrophages in the innate immune responses, which might play central roles in the development of AOSD and MAS.

## Bird’s eye view of the pathogenic process of AOSD

**Table 1** summarizes the pathogenic process of AOSD including the genetic background, triggers, innate and acquired immune systems, high production of ferritin, and proinflammatory cytokines.

**Table 1:** Pathogenic process of AOSD.

Process	Factors	Pathogenic roles and clinical association	References
Genetic background	HLA class I (HLA-B17, B18, and B35) HLA class II (HLA-DR2, DR4, DRB1*12, and DRB1*15)	Disease susceptibility	(10, 11)
	HLA-Bw35 and HLA-DRB1*14 HLA-DRw6 HLA-DRB1*1501 (DR2) and HLA-DRB1*1201 (DR5) HLA-DQB1*0602 (DQ1) IL-18 MIF MEFV, TNFRSF1A LILRA3	Mild, self-limiting disease Joint involvement Chronic disease course Chronic and systemic disease Higher production of IL-18 Higher production of MIF, liver dysfunction Disease susceptibility, severe disease Disease susceptibility, inducing formation of NETs	(12–14) (15) (16) (17) (18)
Triggers	Viruses (rubella, measles, echovirus 7, coxsackievirus B4, cytomegalovirus, Epstein-Barr virus, parvovirus B19, hepatitis virus, influenza virus, adenovirus, and human immunodeficiency virus),	PAMPs	(19–22)
	SARS-CoV-2 Bacteria (*Mycoplasma pneumonia*, *Chlamydia pneumonia*, *Yersinia enterocolitica*, *Brucella abortus*, and *Borrelia burgdorferi*)	Macrophage activation PAMPs	(19) (1, 23–25)
	Solid cancers and hematologic malignancies (necrotic tumor cells)	DAMPs	(1, 26, 27)
Innate immune system	Hyperactivated macrophages	Produce proinflammatory cytokines (IL-1β, IL-18, TNFα, and IL-6) and chemokines Enhanced phagocytosis Stimulate the release of ferritin	(19, 28)
	Hyperactivated neutrophils Low activation of NK cells	Release cytokines/chemokines and communicate with macrophages NETs formation MAS pathogenesis	(29, 30) (31–34)
Acquired immune system	Hyperactivated Th1 cells Hyperactivated Th17 cells Low activation of Tregs	Correlation with clinical activity score and serum IL-18 levels IFNγ activates macrophages Correlation with severity score, serum ferritin levels, and proinflammatory cytokines Disease activity affects the stability of Tregs	(28, 35) (36, 37) (38)
High production of ferritin	Increased ferritin from activated macrophages	Stimulate inflammatory pathways, correlation with disease activity	(1, 19, 39–43)
Proinflammatory cytokines	High levels of IL-18 and IL-1β High levels of IL-6 and TNFα High levels of IL-1β and IL-6 Increased IFNγ level compared with IL-18 IL-18 PLAC8 IL-10 IL-38	Systemic disease Arthritis Fever and skin rash Development of MAS Triggers Th1 response inducing the secretion of IFNγ by cytotoxic CD8^+^ and NK cells Suppresses the synthesis of pro-IL-1β and pro-IL-18 *via* enhanced autophagy in monocytes Inhibits the production of IL-1β, IL-6, and TNFα from monocytes induced by IFNγ Inhibits the activation of NLRP3 inflammasome in macrophages	(1, 3, 44, 45) (46) (47) (48, 49)

MIF, macrophage migration inhibitory factor; MEF, Mediterranean fever; TNFRSF1A, tumor necrosis factor receptor superfamily member 1A; LILRA3, gene name for leukocyte immunoglobulin-like receptor A3; NETs, neutrophil extracellular traps; SARS-CoV-2, severe acute respiratory syndrome coronavirus 2; PAMPs, pathogen-associated molecular patterns; DAMPs, damage-associated molecular patterns; MAS, macrophage activation syndrome; Tregs, regulatory T cells; NK, natural killer; PLAC8, placenta-specific 8; NLRP3, nucleotide-binding oligomerization domain, leucine-rich repeat, and pyrin domain 3.

### Genetic background

A genetic association between AOSD and human leukocyte antigen (HLA), including both HLA class I (HLA-B17, B18, and B35) and HLA class II (HLA-DR2, DR4, DRB1*12, and DRB1*15), has been reported (10, 11). Moreover, some associations of the HLA type with the clinical characteristics of AOSD, such as HLA-Bw35 and HLA-DRB1*14 with a mild, self-limiting disease; HLA-DRw6 with joint involvement; HLA-DRB1*1501 (DR2) and HLA-DRB1*1201 (DR5) with a chronic disease course; and HLA-DQB1*0602 (DQ1) with chronic and systemic disease, have also been shown (12–14). Interestingly, polymorphisms in both the *IL-18* gene and the macrophage migration inhibitory factor (*MIF*) gene have been shown to contribute to the disease susceptibility *via* higher production of IL-18 and MIF (15, 16). For known hereditary periodic fever syndrome genes, a previous study showed that association with 3 rare variants in the Mediterranean fever (*MEFV*) gene, which encodes pyrin, and mutations in the tumor necrosis factor receptor superfamily member 1A (*TNFRSF1A*) gene were significant and that these genetic factors were associated with a severe disease course of AOSD (17). More recently, functional leukocyte immunoglobulin-like receptor A3 (LIR-A3; gene name *LILRA3*) has been identified as a novel genetic risk factor for the development of AOSD, and functional LIR-A3 might play a pathogenic role by inducing formation of neutrophil extracellular traps (NETs) (18).

### Triggers

A large number of viruses, including the rubella, measles, echovirus 7, coxsackievirus B4, cytomegalovirus and Epstein-Barr, parvovirus B19, hepatitis, influenza, adenovirus, and human immunodeficiency viruses, have been suggested to trigger AOSD pathogenesis *via* activation of the aberrant response of the immune system as so-called pathogen-associated molecular patterns (PAMPs) (19–22). More recently, attention has been focused on an association with severe acute respiratory syndrome coronavirus 2 (SARS-CoV-2) and the common features of macrophage activation in coronavirus disease 2019 (COVID-19) and AOSD (19). In addition, some bacteria such as *Mycoplasma pneumoniae*, *Chlamydia pneumoniae*, *Yersinia enterocolitica*, *Brucella abortus*, and *Borrelia burgdorferi* have also been considered to be involved (1, 23–25). Other than these infectious pathogens, solid cancers and hematologic malignancies have been proposed as possible triggers of AOSD (1, 26). Damage-associated molecular patterns (DAMPs) typically released by necrotic tumor cells are recognized by innate immune receptors such as toll-like receptors (TLRs) and could stimulate innate immune responses within the tumor immune microenvironment (27), which might contribute to the development of AOSD. However, no disease-specific unique pathogenic trigger has been clearly identified, suggesting the possibility that multiple environmental triggers play a role in AOSD (1).

### Innate immune systems

Activation of innate immune cells including macrophages and neutrophils plays a major pathogenic role in the development of AOSD (1). The activation mechanisms of monocytes/macrophages will be described in a later section. Hyperactivated macrophages could produce proinflammatory cytokines such as IL-1β, IL-18, TNFα and IL-6, as well as chemokines; enhanced phagocytosis; and stimulated the release of ferritin (19, 28). As for neutrophils, interaction between various PAMPs and DAMPs and a variety of specific receptors including pattern-recognition receptors (PRRs), such as TLRs and C-type lectin domain family 5-member A (CLEC5A), and Fc receptors on neutrophils promote neutrophil recruitment and activation (29). Activated neutrophils release more cytokines/chemokines and communicate with macrophages in the innate immune system. Neutrophil activation and mediator release form a positive-feedback loop that enhances neutrophil recruitment and amplifies inflammatory responses, acting as an axis of pathogenesis in AOSD (29). A previous study demonstrated that IL-18, a pivotal cytokine of AOSD, induces NETs by enhancing calcium influx into neutrophils (29). The protein components of NETs, like S100 proteins, act as ligands of TLR4 or receptors for AGEs (advanced glycation end products) (RAGE) to accelerate neutrophils and activate the release of proinflammatory cytokines, contributing to the systemic inflammation of AOSD (30). Thus, NETs formation can promote cytokine storms by linking neutrophils and macrophages, as described in a later section (29).

Contrastively, it was reported that the cytotoxic function of natural killer (NK) cells was defective in AOSD patients (31). The proinflammatory cytokines produced during AOSD, mainly IL-18, have been reported to decrease NK cell activity (32, 33). Importantly, impairment of NK cell cytotoxicity has been shown to play some roles in MAS pathogenesis (34).

### Acquired immune system

A previous report showed that interferon (IFN) γ-producing helper T (Th) cells and the Th1/Th2 ratio in peripheral blood were significantly higher in patients with AOSD than in healthy controls (HCs) and that the percentages of IFNγ-producing Th cells and the Th1/Th2 ratio in peripheral blood correlated significantly with the clinical activity score and serum IL-18 levels in patients with AOSD (35). Importantly, IFNγ is known to increase the production of cytokines and chemokines, phagocytosis, and the intracellular killing of microbial pathogens by macrophages (28). Moreover, high frequencies of circulating Th17 cells were reported in active AOSD patients and correlated with the severity score, serum ferritin levels, and proinflammatory cytokines including IL-1β, IL-6, and IL-18 (36, 37). In contrast, a recent study clarified that the proportion of regulatory T cells (Tregs) was significantly lower in patients with acute AOSD patients than in the HCs and that the expression levels of IFNγ, IL-17, and IL-4 in Tregs were significantly increased, whilst the suppressive function of Tregs was impaired in patients with acute AOSD (38). These results suggested that the disease activity might affect the stability of Tregs in AOSD (38).

### High production of ferritin

Ferritin is an intracellular iron storage protein including 24 subunits: heavy (H) subunits and light (L) subunits, according to their molecular weight (1). It is well known that ferritin is a characteristic mediator of AOSD (19). In AOSD, H-ferritin and the number of macrophages expressing H-ferritin have been shown to be increased, suggesting a pathogenic role (39–42). Importantly, elevated H-ferritin expressions in the lymph nodes and skin were correlated with the severity of AOSD (41, 42). Ferritin synthesis is regulated, in addition to iron availability, by different cytokines such as IL-1β and IL-6, which are overexpressed in AOSD patients (1, 19). Moreover, ferritin can stimulate inflammatory pathways to amplify the inflammatory process, supporting a hypothesis that ferritin may not only act as a bystander of the acute-phase reaction (43). Thus, increased production of ferritin from macrophage activation might correlate with the disease activity of AOSD and might serve as an activity indicator of this disease (19).

Heme oxygenase-1 (HO-1), an inducible heme-degrading enzyme, is expressed by macrophages and endothelial cells in response to various stresses. Interestingly, it was reported that among patients with hemophagocytic syndrome (HPS) and AOSD, serum HO-1 correlated closely with serum ferritin but not CRP or lactate dehydrogenase (LDH) levels (50). These findings indicated an association between HO-1 and hyperferritinemia in patients with HPS and AOSD.

Moreover, several isoforms of ferritin have been described, one of which is glycosylated ferritin. A previous report revealed that glycosylated ferritin was significantly lower in AOSD patients than in the control patients including infection, liver disease, systemic inflammatory disease, fever of unknown origin, and neoplasia. The study concluded that increased ferritin and decreased glycosylated ferritin levels could be powerful diagnostic markers of AOSD (51).

### Proinflammatory cytokines

The immune system activation described above leads to the production of several proinflammatory cytokines including IL-1β, IL-18, TNFα, IL-6, IFNγ, and IL-17 from activated macrophages, neutrophils, and Th1 and Th17 cells (1). Importantly, different cytokine profiles might be responsible for the distinct clinical manifestation of AOSD (3). For example, high levels of IL-18 and IL-1β are detected in systemic disease; high levels of IL-6 and TNFα, in arthritis; and high levels of IL-1β and IL-6, in fever and skin rash, respectively (1, 3). Regarding MAS, one of the most severe complications of AOSD, a previous study of sJIA showed that an increased plasma IFNγ level in comparison with IL-18 might raise suspicion about the development of MAS in sJIA (44). In addition, IL-6 has also been associated with MAS (3). Regarding the association between IL-18 and IFNγ, IL-18 might also trigger a Th1 response, thereby inducing the secretion of IFNγ by cytotoxic CD8^+^ and NK cells (45). Although these correlations between the cytokine profiles and clinical features of AOSD might be clinically informative, serum cytokines are not routinely examined and might not widely affect our daily practice of individual AOSD patients (3).

## Activation mechanisms of monocytes/macrophages in AOSD

**Figure 1:**
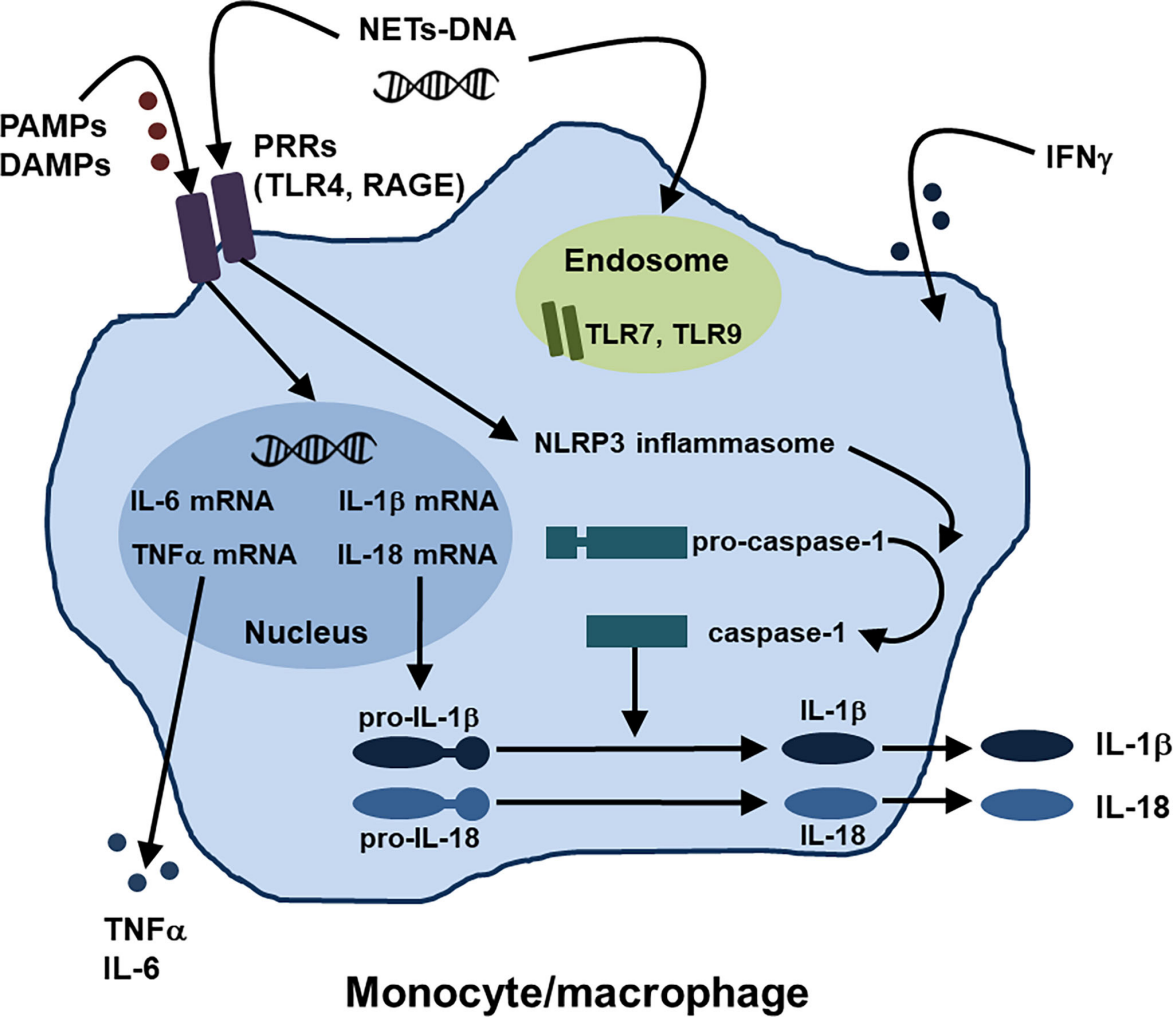
Activation mechanisms of monocytes/macrophages in AOSD. Regarding the activation mechanisms of monocytes/macrophages in AOSD, the PAMPs and DAMPs-PRRs axis and NETs-DNA have been identified, in addition to IFNγ stimulation. NETs, neutrophil extracellular traps; PAMPs, pathogen-associated molecular patterns; DAMPs, damage-associated molecular patterns; PRRs, pattern recognition receptors; TLR, toll-like receptor; receptor for AGEs (advanced glycation end products) RAGE; NLRP, nucleotide-binding oligomerization domain, leucine-rich repeat, and pyrin domain.

In innate immunity, activation of monocytes/macrophages might play central roles in the development of AOSD and MAS, as described above. Regarding the activation mechanisms of monocytes/macrophages in AOSD, several pathways such as the PAMPs and DAMPs-PRRs axis and NETs-DNA have recently been identified (1, 19), in addition to type II IFN stimulation (28). Moreover, several inhibitory molecules that work against activation of monocytes/macrophages have been also reported. **Figure 1** summarizes the activation mechanisms of monocytes/macrophages in AOSD.

### mRNA expression pattern in monocytes from active AOSD patients and identification of PLAC8 as an active-AOSD-specific highly expressed gene which has suppressive ability on the synthesis of pro-IL-1β and pro-IL-18 via enhanced autophagy

We previously compared the gene expression pattern of peripheral monocytes of active-AOSD, inactive-AOSD patients (the same patients in the active and inactive phases), and HCs by DNA microarray to identify genes specifically associated with the active phase of the disease (46). The gene expression patterns in the 3 groups (active-AOSD, inactive-AOSD, and HC) showed distinct clusters specific to each group in principal components analysis (PCA). We identified 68 genes as active-AOSD-specific highly expressed genes, and focused on placenta-specific 8 (*PLAC8*) among them. Importantly, the *PLAC8* mRNA expression levels were significantly higher in the active-AOSD patients than in the HCs or the inactive-AOSD patients, as well as than in patients with rheumatoid arthritis (RA), Sjögren syndrome (SS), systemic lupus erythematosus (SLE), or polymyositis (PM)/dermatomyositis (DM), indicating that the upregulation of *PLAC8* mRNA in monocytes was specific in the patients with active-AOSD. Interestingly, in patients with AOSD, the expression of *PLAC8* mRNA in peripheral monocytes was significantly correlated with serum CRP and ferritin levels. Moreover, serum IL-1β and IL-18 levels, but not IL-6 and TNFα levels, significantly correlated with *PLAC8* mRNA expression levels in the AOSD patients. Thus, these results suggested that the expression levels of *PLAC8* mRNA in peripheral monocytes are an activity or severity marker for AOSD (46).

**Figure 2:**
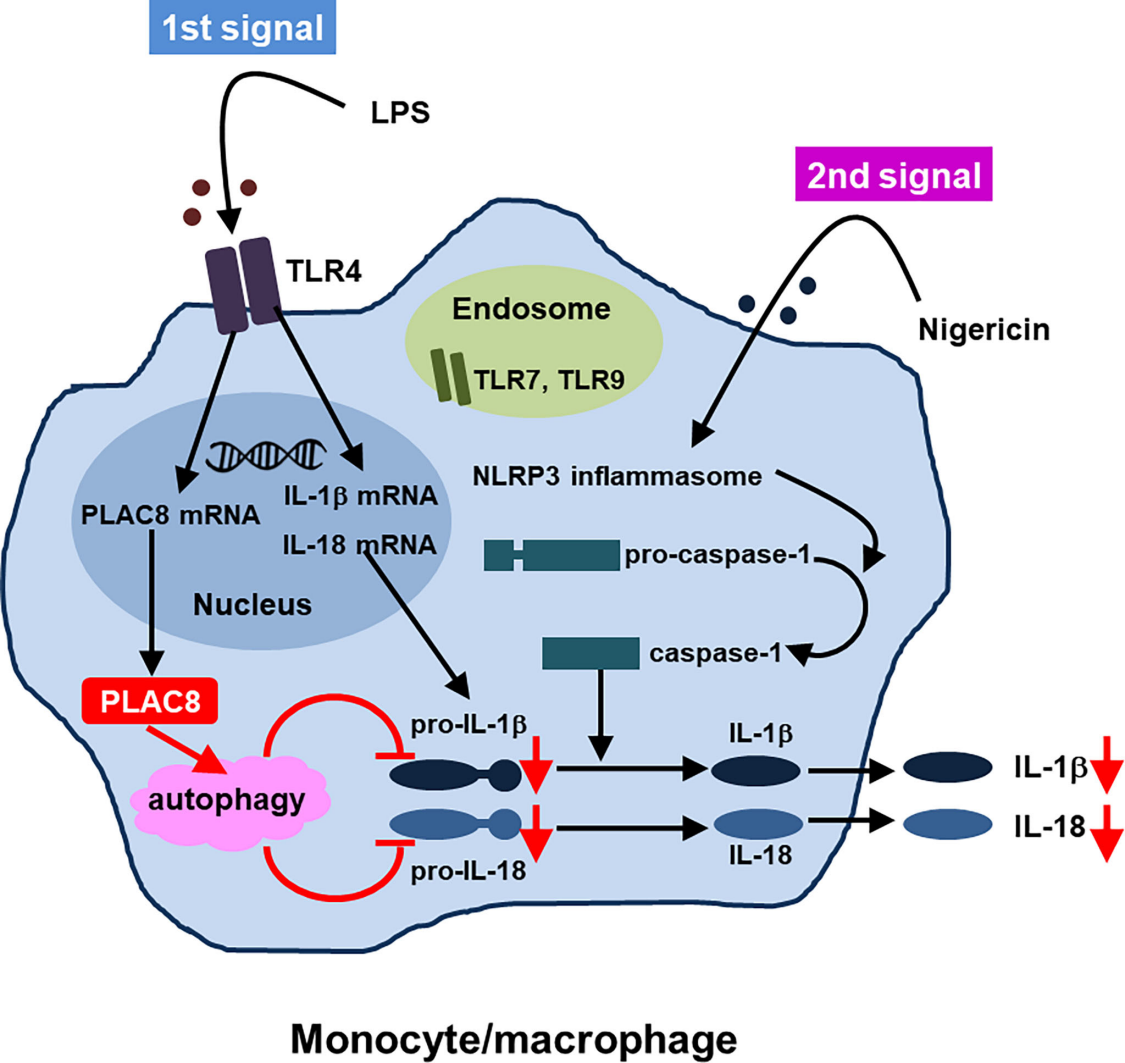
Proposed function of PLAC8 in IL-1β and IL-18 production (reference 46, modification)A schema illustrating how PLAC8 suppresses IL-1β and IL-18 production *via* enhancement of autophagy. Two steps might be needed for inhibition of IL-1β and IL-18 production by PLAC8 in primary monocytes. The first step is the upregulation of PLAC8, pro-IL-1β and pro-IL-18 in monocytes through LPS stimulation. The second step is the inhibition of pro-IL-1β and pro-IL-18 through the enhancement of autophagy by upregulated PLAC8, which is independent of caspase-1.

Regarding the function of PLAC8, we indicated that upregulated PLAC8 acted on the synthesis of inactive precursors of IL-1β and IL-18 and suppressed the production of IL-1β and IL-18 through enhanced autophagy which was independent of caspase-1. Thus, PLAC8 seems to be a regulatory molecule in AOSD. **Figure 2** summarizes the proposed suppressive function of PLAC8 in IL-1β and IL-18 production (46).

### PAMPs and DAMPs-PRRs axis

Monocytes/macrophages could be activated through the recognition of various PAMPs and DAMPs by different types of PRRs such as TLRs, resulting in activation of inflammasomes responsible for pro-IL-1β and pro-IL-18 activation (1, 19). The nucleotide-binding oligomerization domain, leucine-rich repeat, and pyrin domain (NLRP) can form multimeric protein complexes in response to stimuli (19). NLRP3 inflammasomes trigger caspase-1 activation to convert pro-IL-1β and pro-IL-18 into mature IL-1β and IL-18, which contribute to the pathogenesis of AOSD (1, 19).

As for the roles of TLRs and their ligands in activation of monocytes/macrophages in AOSD, interaction between various DAMPs and TLRs, including S100 proteins with TLR4, high mobility group box-1 (HMGB1) with TLR4 and RAGE, and nucleic acids with TLR7, has been identified (19, 52).

### NETs-DNA

As described in the innate immune system section, a previous study demonstrated that IL-18, a pivotal cytokine of AOSD, induces NETs by enhancing the calcium influx into neutrophils (29). Interestingly, Hu et al. showed that NETs-DNA complexes were significantly increased in the circulation of patients with AOSD when compared with that of HCs and that NETs-DNA from AOSD patients activated macrophages and increased the expression of IL-1β, IL-6, and TNFα *via* activation of the NLRP3 inflammasome (53). The authors hypothesized that AOSD neutrophils spontaneously release NETs-DNA, leading to an enhanced proinflammatory potential mainly *via* TLR9 (53). These findings indicated a novel link between neutrophils and macrophages by NETs formation in AOSD.

### Type II IFN stimulation

IFNγ, type II IFN, would be the prototypic “macrophage-activating factor” which could increase cytokine and chemokine production, phagocytosis, and the intracellular killing of microbial pathogens by macrophages, thus playing important pathogenic roles in the development of AOSD and MAS (28, 54)

### Inhibitory effect of IL-10 and IL-38 on proinflammatory cytokine production

A recent study showed that peripheral monocytes expressed IL-10 receptors as well as IL-6 receptors and gp130 (47). As expected, IFNγ enhanced the expression levels of proinflammatory cytokines including IL-1β, IL-6, and TNFα from monocytes (47). Interestingly, IL-10 clearly inhibited the production of these cytokines induced by IFNγ stimulation (47). Thus, IL-10 seems to have an inhibitory effect on proinflammatory cytokine production by monocytes.

IL-38 is a new member of the IL-1 family with multiple functions involved in infection and immunity. A recent study revealed that LPS upregulated IL-38 and its receptor IL-36 receptor, and IL-38 shifted macrophages from a M1 to M2 phenotype, as well as IL-38 dampened LPS induced activation of NLRP3 inflammasome in mouse peritoneal macrophages (48). Thus, IL-38 can significantly inhibit the activation of NLRP3 inflammasome, resulting in a potent anti-inflammatory activity (49).

## Conclusion

Various factors including the genetic background, triggers, innate and acquired immune systems, high production of ferritin, and proinflammatory cytokines could contribute to the pathogenic process of AOSD. Among innate immunity, activation of monocytes/macrophages might play central roles in the development of AOSD and MAS. Regarding the activation mechanisms of monocytes/macrophages in AOSD, several pathways such as the PAMPs and DAMPs-PRRs axis and NETs-DNA have been identified, in addition to type II IFN stimulation. We identified PLAC8 as a specifically increased molecule in monocytes of active AOSD, which correlated with serum levels of CRP, ferritin, IL-1β, and IL-18. Interestingly, PLAC8 could suppress the synthesis of pro-IL-1β and pro-IL-18 *via* enhanced autophagy; thus, PLAC8 seems to be a regulatory molecule in AOSD. These findings related to the activation mechanisms of monocytes/macrophages could shed light on the pathogenesis of AOSD and thus lead to the development of a novel therapeutic strategy for the disease.

## Author contributions

Each author took part in the design of the study, collection of the data, and writing of the manuscript, and all agree to accept equal responsibility for the accuracy of its contents of this paper. All authors contributed to the article and approved the submitted version.

## Funding

This work was supported by the Research Program for Intractable Diseases, Health and Labor Sciences Research Grants from the Ministry of Health, Labor and Welfare, Japan and Grants-in-Aid for Scientific Research from the Ministry of Education, Culture, Sports, Science and Technology and Japan Society for the Promotion of Science.

## Acknowledgments

We thank Flaminia Miyamasu, Medical English Communications Center, University of Tsukuba, for grammatical editing.

## Conflict of interest

The authors declare that the research was conducted in the absence of any commercial or financial relationships that could be construed as a potential conflict of interest.

## Publisher’s note

All claims expressed in this article are solely those of the authors and do not necessarily represent those of their affiliated organizations, or those of the publisher, the editors and the reviewers. Any product that may be evaluated in this article, or claim that may be made by its manufacturer, is not guaranteed or endorsed by the publisher.
